# Government and farmer responses to the fall armyworm outbreak in mainland Southeast Asia

**DOI:** 10.3389/finsc.2024.1455585

**Published:** 2025-01-22

**Authors:** Eiichi Kusano, Nipon Poapongsakorn, Urairat Jantarasiri, Kamphol Pantakua, Cuong H. Tran, Khin Akari Tar, Thong Kong, Viengsavanh V. Phimphachanhvongsod, Youichi Kobori

**Affiliations:** ^1^ Social Sciences Division, Japan International Research Center for Agricultural Sciences, Tsukuba, Japan; ^2^ Modern Agriculture Policy, Thailand Development Research Institute, Bangkok, Thailand; ^3^ Good Regulatory Policy, Thailand Development Research Institute, Bangkok, Thailand; ^4^ Faculty of Accounting and Business Management, Vietnam National University of Agriculture, Hanoi, Vietnam; ^5^ Freelance Researcher, Yangon, Myanmar; ^6^ Programme, INGO Forum Myanmar, Yangon, Myanmar; ^7^ Faculty of Agro-Industry, Royal University of Agriculture, Phnom Penh, Cambodia; ^8^ Planning and Cooperation Division, National Agriculture and Forestry Research Institute, Vientiane, Lao People's Democratic Republic; ^9^ Crop, Livestock, and Environment Division, Japan International Research Center for Agricultural Sciences, Tsukuba, Japan

**Keywords:** Asia-Pacific region, corn, cost-efficiency, integrated pest management (IPM), maize, *Spodoptera frugiperda*

## Abstract

**Introduction:**

Native to the Americas and highly polyphagous, the fall armyworm (FAW), *Spodoptera frugiperda* (J.E. Smith) (Lepidoptera: Noctuidae) has garnered attention for causing significant damage, primarily to maize.

**Methods:**

This study synthesizes FAW emergence, government responses, and farmer reactions in mainland Southeast Asia (MSEA), and assesses the feasibility of government-recommended measures in terms of efficacy and cost-efficiency.

**Results:**

From late 2018 to the rainy season of 2019, FAW infestations extensively emerged in MSEA maize fields. MSEA governments promptly issued strategies and guidelines through plant protection divisions/departments, which involved international organizations, foreign governments, and private web portals. Alongside the foliar application of emamectin benzoate (EMB), which is the most frequently mentioned method, MSEA governments advocated for integrated pest management (IPM)-oriented approaches. These include application methods of chemical insecticides, use of host plant resistance, biological control, cultural and interference methods, and local measures aimed at reducing chemical usage. Despite comprehensive recommendations, maize farmers primarily rely on EMB foliar treatment for FAW control.

**Discussion:**

We highlight the need for further research and dissemination regarding the widely accepted foliar application of chemical insecticides, specifically in relation to human safety, improvements in application technology, and clear guidelines for large-scale outbreaks. On the other hand, the concentrative foliar application of chemical insecticides raises concerns about resistance evolution. Alternatives to foliar treatment with chemical insecticides, mainly EMB, such as seed treatment with diamides and neonicotinoids, genetically modified maize seeds approved only in Vietnam, and biological control, have demonstrated efficacy. Seed treatment provides cost and labor benefits for early-stage FAW infestation prevention. Validation of natural enemy rearing costs may prove advantageous as preliminary estimates suggest they could be relatively low. Not all strategies recommended by the government or widely discussed are necessarily relevant at farm-level. This study provides the following suggestions for the proposal of more acceptable strategies. 1) Studying the actual responses of governments and farmers with special emphasis on cost efficiency; 2) Making alternatives to EMB foliar application more cost-effective inclusive of the cost of labor; and finally, 3) Verifying the effectiveness of the alternative techniques.

## 1 Introduction

The fall armyworm (FAW), *Spodoptera frugiperda* (J.E. Smith) (Lepidoptera: Noctuidae), which is highly polyphagous and originates from the Americas, is notorious for causing significant damage to maize crops (1, 2). First observed outside the Americas in West Africa in 2016, it quickly spread to Asia. Initial sightings were reported in Karnataka, India, in May 2018, followed by subsequent reports throughout the country (2–6). In Southeast Asia, FAW was reported in Myanmar in July 2018. By May 2019, it had spread to all mainland regions, including Malaysia, and later reached Indonesia and the Philippines by late 2019 (7–19).

In response to FAW’s rapid spread in Southeast Asia, international organizations and the Association of Southeast Asian Nations (ASEAN) swiftly provided basic guidelines for integrated pest management (IPM). The Food and Agriculture Organization of the United Nations (FAO) organized consultative meetings in Bangkok in March 2019 and a regional workshop in Kunming City, China, in November of the same year, ultimately emphasizing the importance of IPM strategies (20, 21). Additionally, the ASEAN Action Plan on Fall Armyworm 2020–2025, developed by Grow Asia in collaboration with the Vietnamese Ministry of Agriculture and Rural Development (MARD), the ASEAN Secretariat, and the FAO Regional Office for Asia and the Pacific, was approved by ASEAN in October 2020, with the aim of promoting sustainable and cost-effective IPM across the region (22, 23).

Recent advancements in studying FAW management techniques, biology, and ecology have underscored the importance of IPM strategies. Guidelines and review studies on FAW management strategies across Asia share similar recommendations, which include: monitoring FAW infestations and controlling pests based on action thresholds to avoid the excessive use of chemical insecticides; considering efficiency, safety, and rotation use for resistance management when using chemical insecticides; exploring alternative methods to reduce chemical insecticide use, such as biological control, including the investigation of indigenous natural enemies and their release; using microbial insecticides, especially *Bacillus thuringiensis* (*Bt*) and botanical insecticides; employing agroecological or cultural controls, such as early planting and intercropping; and utilizing resistant cultivars, including genetically modified (GM) crops (2, 6, 24–27).

Southeast Asian governments have prioritized IPM strategies for FAW management. However, two critical issues need to be addressed. First, the specific content and implementation status of the relevant policies remain fragmented, thus complicating effective control given FAW’s significant mobility. A comprehensive understanding of management strategies across the region is essential for effective control (23, 24). Second, the farm-level feasibility of the comprehensive government recommendations aimed at promoting IPM needs to be verified. In Africa, the use of chemical insecticides remains dominant despite the recommendations for alternative practices, which suggests that similar challenges could arise in Southeast Asia (28–30). Comprehensive strategies are proposed in guidelines and review studies targeting FAW management in Asia. However, this does not necessarily mean those strategies are easy to adopt at the farm level. These recommendations are largely based on laboratory and field trials (2, 6, 25, 27), as well as consultations with stakeholders responsible for pest management in various Asian countries (26), rather than on actual farm-level adoption. Amid this context, the cultivation area of GM maize has been steadily expanding in Vietnam and the Philippines, thereby suggesting the utility value of this technology at the farm level (31, 32).

This study analyzes FAW management practices implemented by governments and farmers in mainland Southeast Asia (MSEA), including Myanmar, Thailand, Laos, Cambodia, and Vietnam. We focused on MSEA because it is a geographically contiguous region characterized by consistent climates with low precipitation during winter, specifically tropical savanna and monsoon-influenced humid subtropical climates (33). The predominance of climates with clearly defined dry seasons distinguishes MSEA from maritime Southeast Asian countries, such as Malaysia, Indonesia, and the Philippines, which are also part of ASEAN but are predominantly characterized by tropical rainforest climates (33). Differences in seasonal precipitation patterns can influence maize cultivation and FAW outbreaks (34). Therefore, focusing on MSEA as a regional unit is advantageous for ensuring consistency in our discussion.

## 2 Materials and methods

To provide insights for further FAW management strategies, this study outlines the timing of FAW’s first detection and governmental responses in each country, illustrates farmers’ practices to control FAW, and discusses the feasibility of government recommendations, with a focus on efficacy and cost-effectiveness.

### 2.1 Utilizing secondary data to summarize the FAW outbreak and government responses

We utilize secondary data from national government websites, publications, and previous studies to summarize the spread of FAW and governmental responses. These sources help determine the timing and extent of FAW infestation across countries and identify responsible entities and their actions for FAW control. We also summarize the recommendations for FAW control promoted to agricultural producers in each country through brochures, leaflets, websites, and Facebook written in local languages. Following Dent and Binks (35), government-recommended measures in each country are categorized into the following six groups: sampling and monitoring, chemical control, host plant resistance, biological control, cultural and interference methods, and other methods.

### 2.2 Surveying farmers’ reactions to FAW infestation

We conducted a sample survey in Thailand’s primary maize-producing regions in 2021 and qualitative interviews across multiple countries in 2022 to investigate farmers’ practices in mitigating FAW damage.

Conducted from August to September 2021, the Thai survey employed a structured form listing FAW management methods, thus allowing for multiple selections. Targeted regions included high maize production areas around the ranges separating the central plain and Khorat Plateau. A total of 127 farmers from seven districts participated: Phetchabun (Lom Kao, Chon Daen, and Nong Phai), Lopburi (Chai Badan), and Nakhon Ratchasima (Dan Khun Thot, Pak Chong, and Sung Noen).

In 2022, qualitative interviews were conducted with maize production experts in Myanmar, Laos, and Cambodia to explore FAW management among farmers. Interviews were carried out in February 2022 for Myanmar and Cambodia and in October 2022 for Laos at various locations: Taunggyi District, Shan State, Myanmar (three maize farmers, one input dealer, and one researcher from a research farm station under the Department of Agricultural Research [DAR]); Xaythany and Naxaithong Districts, Vientiane Prefecture, Laos (three researchers at the Rice Research Centre and one farmer); Lvea Aem District, Kandal Province, and Tbong Khmun District, Tbong Khmun Province, Cambodia (one district officer, one import supplier, and two farmers each).

### 2.3 Surveying the cost structure of FAW control among maize farmers

To determine the cost structure of pest management among maize farmers in each country in 2022, structured interviews were conducted from October 2022 to March 2023. We targeted farmers heavily reliant on maize for their livelihoods, with cultivation areas ranging from 1.6 ha to 16.0 ha. The survey included 19 farmers across all MSEA countries, which resulted in valid responses from 14 farmers in four countries, excluding Laos. Valid responses were obtained from regions including Shan State (Myanmar), Son La Province (Vietnam), Lopburi Province (Thailand), and Kandal Province (Cambodia), which are known for substantial maize production.

Data on tasks for both dry and wet season maize production in 2022 were collected, including land preparation, seed treatment, sowing, fertilizer application, weed control, insect control, harvesting, and post-harvesting. A total of two cases exist for the work at each stage: family labor and outsourcing to others. For family labor, we estimated the opportunity cost based on the labor hours and the minimum wage. For outsourced work, the cost of outsourcing was calculated as the amount paid to others, which may include rental fees for fixed assets such as equipment and machines. When farmers owned fixed assets themselves, we estimated the depreciation cost using the straight-line method based on the number of assets, year of purchase, useful life, and purchase price. If the same fixed assets were used for multiple tasks, the depreciation cost was allocated based on the time spent on each task. If fixed assets were also used for other crops, the depreciation cost for maize was estimated by applying the proportion of the maize cultivated area. Fuel costs were determined from the amount of fuel used and its price when operating each equipment or machine. When a single piece of equipment was used for multiple tasks, such as sowing and fertilizer application, the fuel costs were allocated accordingly. For inputs such as insecticides, we collected information on their type, quantity, and price. We also gathered other information such as land rent, irrigation costs, and the minimum wage. Additionally, information on the types of pests in maize production and their subjective impact on yield was collected using a five-point scale ranging from none to catastrophic damage. For maize sales, we obtained data on the moisture content of the grain, sales volume, and sales price, which varied for each farmer.

Maize production costs were categorized into explicit (e.g., input goods, outsourcing, farm machinery fuel, land rent, and irrigation) and implicit (e.g., farm machinery and equipment depreciation, opportunity cost of family labor) costs for each farmer. To confirm the generalizability of results, we compared these with studies involving larger sample sizes. Moreover, we summarized the costs of input goods and their application for pest extermination and prevention for each farmer.

## 3 Results

### 3.1 Timings of FAW’s first detection and government responses in MSEA

#### 3.1.1 First detection timings of FAW

Between 2018 and 2019, severe damage caused by FAW was initially observed in southern Myanmar, which subsequently spread to the northeastern regions (7, 24). In July 2018, FAW sightings were reported at the DAR Tetkone experimental field in Nay Pyi Taw Region, followed by sightings in Nweyit village, Tatkon township, in November of the same year, as documented by a DAR and Yunnan Academy of Agricultural Sciences survey [**Table 1**; (7)]. Further investigations by the Department of Agriculture (DOA) and Plant Protection Division (PPDiv) during 2018 and January 2019 identified FAW infestations in several regions, including Mandalay, Ayeyawaddy, Mon, Kachin, Sagaing, and East Shan (62–64).

**Table 1:** Timing of initial FAW detection and subsequent intensive infestation areas (1,000 ha) during the 2018–2019 outbreaks in MSEA.

Country	First detection period	FAW infestation			Cultivation area	
		Data period	Infested area	Maize cultivated area	Maize (grain)	Maize (vegetable)
Myanmar	Jul 2018	By the end of 2018	65	N/A	520	(369^h^)
		Oct 2018–Feb 2019^b^	59	N/A		
		May 2019–Oct 2019^b^	45	N/A		
Thailand	Dec 2018	Aug 15–21, 2019^c^	236	873	1,124	65
Laos	Jan 2019	By Jul 2019	40	104	124	28
		By Aug 2, 2019	48	N/A		
Cambodia	May 2019	By Jun 11, 2019	11	N/A	200	22
Vietnam	Mar 2019^a^	Jul 19–27, 2019^c^	18	(335^e^)	987	N/A
		By Aug 9, 2019	42	N/A		
For reference						
Malaysia	Feb 2019	By Sep 2019	0.25	N/A	7.3	7.6
Indonesia	Mar 2019	Apr 2018–Sep 2019	7^d^	N/A	(2,338^g^)	(121^i^)
		Oct 2019–Mar 2020	12^d^	N/A		
Philippines	Jun 2019	Oct 2019	0.22	N/A	1,415	1,101
		Jun 2020	6	(679^f^)		

a First detection period after MARD initiated vigilance for FAW in February 2019.

b Months corresponding to the periods originally labeled as Winter 2018 and Monsoon 2019 were specified based on DAR (36).

c Reported values for the weeks surrounding the peak infestation period of FAW.

d The infested area includes the total for FAW and *Spodoptera litura* (Fabricius) (Lepidoptera: Noctuidae), but the source suggests that FAW infestations were predominant (37, 38).

e Reference value for July 16–22, 2020.

f Area of standing maize crops as of September 15, 2020.

g Reference value for 2020.

h Reference value for 2016.

i Reference value estimated by FAO (39). The cultivation area represents the sown area for maize (grain and vegetable) in MSEA counties and maize (vegetable) in Malaysia and refers to the harvested area for others. The cultivation area values are from 2019 unless otherwise noted. Infested areas are strictly incomparable between countries due to potential differences in outbreak definitions and the timing of infested area measurements. The term maize (grain) primarily refers to yellow maize used as animal feed. The FAW-infested area in Vietnam by August 9, 2019, is the sum of values by province, with province-specific values estimated through numerical conversion of the map illustrating the cumulative infested area up to July 25, 2019, from USDA (40), relying on local reports from the MARD. The information has been interpolated and updated using media reports until August 9, 2019. “N/A”, not available. Sources: First detection period (7–19). FAW infestation ((9, 18, 23, 37, 38, 40–51; DOAE Myanmar 2019, unpublished data). Cultivation area (39, 52–61).

FAW was first detected in Thailand, Laos, and Cambodia between December 2018 and May 2019 (**Table 1**). Thailand remained vigilant against the invasion from neighboring provinces in Myanmar, including Mae Hong Son, Tak, Chiang Mai, and Chiang Rai, after August 2018 (65). In December 2018, FAW was confirmed in samples collected from maize in Kanchanaburi and Tak Provinces (8), which led to subsequent nationwide confirmations. In January 2019, FAW was first detected in Sendin Village, Naxaithong District, which is near the Thai border in Laos (9, 10). A team comprising individuals from the International Maize and Wheat Improvement Center (CIMMYT), Lao Upland Rural Advisory Service (LURAS), and the provincial Agriculture and Forestry Office confirmed an outbreak of FAW in Xiengkhuang at the end of May and beginning of June 2019 (66). A government notice on June 6 reported a widespread FAW infestation in Sayaboury, Vientiane Province, Udomsai, Borikhamxay, and Savannakhet (9). According to LURAS (10), FAW had been recorded in all provinces by June 2019. In Cambodia, FAW was discovered in a maize field in the Malai District in Banteay Meanchey, which neighbors Thailand, in May 2019 (11, 67). By June 11, 2019, the Cambodian Ministry of Agriculture, Forestry, and Fisheries (MAFF) had reported FAW damage in Pailin, Battambang, Banteay Meanchey, and Tbong Khmum (43, 67). By 2021, FAW had spread to nearly all production regions in Cambodia, although information on affected provinces was limited (68, 69).

In Vietnam, FAW was initially detected in Phu Dien, Hanoi, on carpetgrass in 2008. Its presence was confirmed 11 years before the official announcement of the 2019 outbreak (70, 71). However, following warnings from the FAO and Commonwealth Agricultural Bureaux (CABI) (40, 71), the Plant Protection Department (PPDpt) under the MARD issued an official announcement (No. 351/BVTV-TV) regarding vigilance against FAW as a new pest on February 19, 2019. In early March 2019, the first FAW detection after heightened vigilance occurred in a maize field in Dong Nai Province in southern Vietnam [**Table 1**; (72)]. Damage caused by FAW was reported between March and April in major maize production areas, including the Red River Delta, northern mountainous regions, and the north-central region (12). Then, in April 2019, the official confirmation of the presence of FAW in Vietnam was announced based on a genetic analysis conducted by CABI (No. 937/BVTV-TV).

#### 3.1.2 Responsible entities and their responses to FAW infestation

In each MSEA country, ministries of agriculture oversee plant protection divisions/departments, which collaborate with international organizations and development agencies to formulate and publish guidelines addressing FAW infestations. Furthermore, private companies contribute to disseminating information in some countries.

In Myanmar, the PPDiv of the DOA under the Ministry of Agriculture, Livestock, and Irrigation (MOALI) released FAW control measures from August 2018 to June 2019, accessible via Green Way (73–75). These measures include weed control, intercropping, potash fertilizer application, handpicking, and ash distribution into maize leaf whorls. Facebook posts attributed to the PPDiv highlighted scouting and control measure selection based on FAW infestation levels, which were confirmed in January and July 2019 (76, 77). The PPDiv (78) issued a booklet emphasizing scouting and incorporating agroecological-based control practices outlined in the IPM guide against FAW in Africa, which was led by the U.S. Agency for International Development (USAID) and CIMMYT (3). Additionally, the FAO and PPDiv (63) issued similar manuals. Additionally, the DAR under MOALI publishes IPM information on FAW through its Facebook page and newspapers (79), aligned with content provided by the PPDiv.

In Thailand, the Plant Protection Promotion and Soil-Fertilizer Management Division (PPSF) under the Department of Agricultural Extension (DOAE) and the Plant Protection Research and Development Office (PPRDO) under the DOA, both under the Ministry of Agriculture and Cooperatives, lead the dissemination of information on FAW ecology and control measures through their respective websites. The PPSF issued pest alerts with FAW control measures from May to July 2019 (80, 81). In July 2019, the PPRDO and DOA published a leaflet and a poster summarizing FAW control measures (82, 83). Similarly, the Nakhon Sawan Field Crops Research Center, which is affiliated with the Field and Renewable Energy Crops Research Institute under the DOA, has provided detailed information on FAW ecology and control methods on its website since October 2020 (84). In 2021, the PPSF organized FAW control measures in maize, which are similar to the FAO’s format (85), in their “Pest Management Decision Guide” (86).

In Laos, the Plant Protection Center under the DOA of the Ministry of Agriculture and Forestry developed a poster summarizing FAW awareness and measures (87). Another valuable resource is the materials from LURAS, which is funded by the Swiss Agency for Development and Cooperation. The project report of LURAS highlighted treatment of Guduchi (*Tinospora cordifolia*) extract and predatory stink bugs for release against FAW on sweet corn, resistant seed selection to minimize pest damage, and forecasting FAW outbreaks for efficient utilization of natural enemies and pathogens (16, 88). Additionally, the government encouraged farmers in Laos to transition from maize cultivation to economically important crops like peanuts and soybeans in heavily affected provinces, including Xayabury, Oudomxay, Huaphan, and Xiangkhouang (89).

In Cambodia, the Plant Protection, Sanitary, and Phytosanitary Department (PPSPD) of the General Directorate of Agriculture (GDA) under the MAFF compiled FAW control measures, which are accessible through the website of the Provincial Department of Agriculture, Forestry, and Fisheries (PDAFF). In June 2019, the MAFF issued a document outlining measures to control FAW across relevant sectors (43). In July 2019, funded by the International Rice Research Institute, the PPSPD released a poster describing specific FAW control methods (90). Local governments in provinces such as Tboung Khmum (91) and Pailin (92) disseminated similar measures through Facebook and government websites.

In Vietnam, the MARD and its agencies, the PPDpt and the National Agricultural Extension Centre (NAEC), consistently published measures to address FAW. These measures are primarily accessible through the NAEC and local governments’ websites. In April 2019, the MARD provided guidance on FAW response to the regional MARD and its affiliated institutes, including the PPDpt and NAEC (93). In May 2019, the PPDpt issued a letter summarizing the physiological and biological characteristics of FAW and IPM for the Provincial MARD (94). In January 2020, the MARD issued a decision providing further details on resistant varieties, seed treatment, adult moth trapping, and conditions for applying chemical insecticides (95, 96). Similar measures were compiled in a leaflet jointly issued by PPDpt and NAEC in July 2019 and August 2021 (97, 98).

#### 3.1.3 Details of FAW management recommendations

MSEA countries recommend not only specific active ingredients for chemical control as part of their FAW management strategies, but also other measures to reduce chemical insecticide usage (**Supplementary Table S1**). Aiming for IPM, these strategies align with the guidelines for FAW in Asia set by USAID, CIMMYT (2), and FAO (26).

Chemical insecticides, particularly emamectin benzoate (EMB), are the most widely mentioned, followed by indoxacarb, chlorantraniliprole, and flubendiamide (**Supplementary Table S1**). Many countries stress the use of the microbial insecticide *Bt* and advocate against continuous use of chemical insecticides with the same active ingredient or Insecticide Resistance Action Committee group for insecticide resistance management (99). Other frequently mentioned methods include insect traps for monitoring, seed treatment with cyantraniliprole, and releasing *Trichogramma* spp, and various natural enemies. Additionally, weeding and handpicking are recommended, although their efficacy warrants further validation (**Supplementary Table S1**).

Notable differences exist among countries’ responses. Myanmar emphasizes various agronomic practices, drawing on Prasanna et al. (3) and Zaois-Tech (100) (63, 78). Thailand provides detailed information on natural enemy release [**Supplementary Table S1** (86)]. Vietnam recommends GM maize, which is approved only in Vietnam within the MSEA, for areas with significant FAW damage, alongside mass trapping using sweet-sour bait for adult control (95). Laos adopted strategies that combine elements of Thailand and Vietnam’s approaches, which include *Bt* spraying, mass trapping, seed treatment, and foliar treatment (87). By contrast, Cambodia recommends unique methods such as trenching along fields, creating artificial habitats for FAW, and sand-diesel sprinkling (90).

Thailand, Vietnam, and Myanmar recommend the control of FAW based on maize growth stages, focusing on measures during the vegetative stage (**Supplementary Figure S1**). Action thresholds for chemical insecticide application are relatively low during emergence to sixth-leaf stages when maize is most vulnerable to FAW (101). Thailand and Vietnam recommend seed treatment using cyantraniliprole for protection during these stages.

The tasseling to kernel blister stages are critical as FAW can significantly impact maize yields (101). However, all countries and organizations exhibited in **Supplementary Figure S1** exhibit caution regarding chemical insecticide use after the tasseling or silk stages. Thailand and Vietnam emphasize diminished effectiveness of insecticide spray once the worm pierces the ear (84, 95). Additionally, smallholders may be exposed to insecticides when spraying overhead onto maize during the tasseling or reproductive stages (63, 102). McGrath et al. (101, 102) recommend using low-toxicity insecticides and wearing personal protective equipment during foliar sprays in this period.

### 3.2 Farmers’ responses to FAW infestation in MSEA

#### 3.2.1 Overview of farmers’ responses

Despite MSEA governments promoting IPM techniques for addressing FAW, foliar treatment using chemical insecticides remains prevalent among farmers. According to our survey of 127 farmers in Central Thailand, the most common response to discovering FAW larvae was the application of chemical pesticides, at 87%, followed by the use of microbial insecticides like *Bt* at 8%, doing nothing at 2%, hand picking at 2%, and never encountering FAW at 2%. Our survey did not observe the use of other methods such as releasing natural enemies. During the pre-planting stage, 95% of farmers reported implementing FAW prevention through plowing as recommended by the Thai government (**Supplementary Table S1**), while 5% did nothing, and none applied seed treatment.

In Vietnam, conventional maize farming in 2018–2019 witnessed extensive insecticide use for pest control, constituting 72% of practices (103). According to Nguyen and Gilleski (104), most provinces followed MARD guidance, with local agricultural agencies leading and farmers adhering to measures such as using suitable maize varieties, including GM maize, surveillance, and employing chemical and biological methods. The area cultivated with GM maize in 2019/2020 is estimated to be around 10% of the total crop area or 100,000 ha (103, 104).

Information from interviews conducted in Cambodia’s Kandal Province and Tbong Khmum Province in 2022 suggests that, despite educational efforts by the GDA, PDAFF, and district officials to promote the use of traps for monitoring, farmers do not consistently adopt this practice. Instead, they prefer to spray pesticides when infestations are observed. Similar findings were observed in Shan State, Myanmar, in 2022, where researchers advocate for IPM usage, but farmers commonly rely on pesticides available at local stores. In Laos, interviews conducted in Vientiane Prefecture in 2022 indicate that government FAW management measures are not widely known, and owing to cost considerations, even pesticide application is not actively pursued.

#### 3.2.2 Cost structure of farmers’ maize cultivation

In 2022, operational costs of maize production in various countries consistently reflected high input goods costs and significant outsourcing or implicit costs for some farmers, which is consistent with prior studies that conducted more extensive sample surveys (**Supplementary Table S2**). Data from the interviews illustrate that revenue and costs in Vietnam tend to be higher in dollar terms compared to other countries. Farmers No. 2 and 3 in Vietnam have notably elevated opportunity costs for family labor, possibly because this study only considers the number of workers and hours worked for estimating opportunity costs without accounting for work intensity. To interpret that work intensity is low may be more accurate, thereby making these values comparable to other farms in Vietnam (No. 1 and 4).

**Figure 1:**
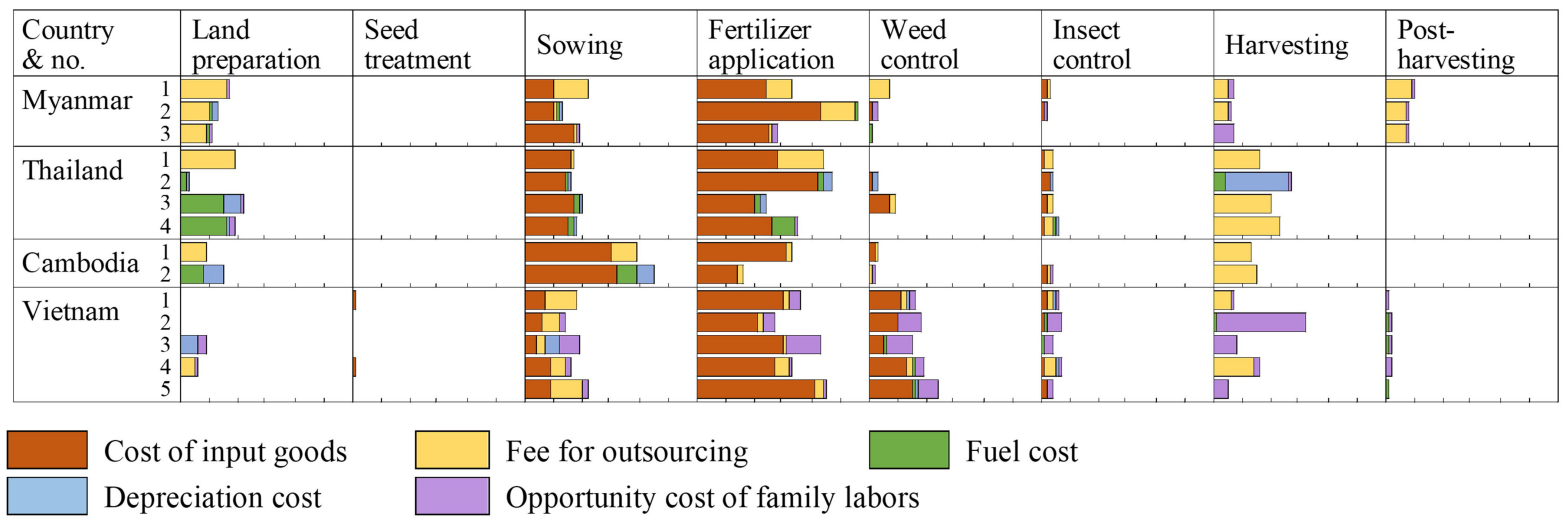
Proportion of production costs for each maize production stage, wet season, 2022 (%). The figure illustrates the various costs as a percentage of total costs for each farmer. The horizontal axis is labeled at 10% intervals, with all categories capped at a maximum of 60%. “Insect control” denotes foliar chemical insecticide treatment. “Post-harvesting” includes de-husking, threshing, drying, and transportation of seeds from the field to the market. “Disease prevention and curing” were not included due to zero responses in the survey. Source: Authors’ surveys in 2022 and 2023.

The cost breakdowns in **Figure 1** reveal that insect control expenses generally account for 5% of total costs for many farms, with constrained spending on insecticides. Fertilizer costs represent a significant portion of expenses among surveyed farmers, while seed costs are substantial, particularly in Cambodia, Thailand, and Myanmar. Outsourcing, fuel costs, and the opportunity cost of family labor tend to be higher for land preparation and harvesting.

These findings align with previous research that highlights substantial input costs for fertilizers, other chemicals (especially herbicides), and seeds, as well as relatively high operating costs or labor inputs associated with harvesting and land preparation (105–111). However, these studies do not address the cost of insect control, ultimately suggesting it may be relatively low.

The relatively low cost of insect control in maize production in MSEA is also supported by national statistics from neighboring countries that are not part of the focus region of the current study. These sources indicate that the average cost of pesticides for insect, disease, and weed control in yellow maize in the Philippines accounted for 4.0% of the total production cost during 2019–2021 (112). In Indonesia, the cost of pesticides for maize was 3.5% in 2017 (113).

#### 3.2.3 Cost of foliar chemical insecticide treatment

Upon reviewing responses to pest damage, farmers clearly perceive pesticide application as effective in mitigating yield losses. Of the 14 farmers interviewed, 12 reported pest damage attributed solely to FAW during wet season cultivation. FAW damage severity was assessed using a five-point scale, with 19 out of 20 cases rated as “none” or “slight” in terms of impact on yield.

The costs for input goods and their application for pest insect control are relatively low. All respondents used foliar treatment with chemical insecticides against FAW infestation (**Supplementary Table S3**), with EMB being the most common active ingredient due to its low application costs. The cost of insecticides with EMB water-dispersible granules ranges from $4.3 to $17.1/ha per application, with a median of $8.9/ha, excluding farmers who applied them to only a portion of cultivated areas. This cost remains consistent even when including other ingredients like indoxacarb and permethrin, and closely align with prices at $7.5–$8.0/ha per application in Asia as reported by Prasanna et al. (114). Survey results indicate a frequency of 1–2 applications per season per farmer, consistent with prior studies that average 1.1–1.4 applications per season for maize in Thailand and Vietnam (103, 111). This frequency was obtained by calculating the average insecticide usage per ha, adjusting it to the 2022 levels using exchange and inflation rates (115, 116), and then dividing by $8.9/ha.

Median insecticide application costs for foliar treatment, including outsourcing, fuel, and depreciation, were $4.6/ha per application. While some farmers incur higher expenses, such as farmer No. 4 in Vietnam, with costs reaching $47.8/ha, the proportion of total costs to total expenses remains low at 4% (**Figure 1**).

#### 3.2.4 Labor burden for insect control

Foliar treatment, particularly with labor-intensive methods such as knapsack sprayers used on larger areas (2 ha and above) (**Supplementary Table S3**), can pose significant labor burdens. Labor requirements for spraying 1 ha with knapsack or stationary engine sprayers vary from 2 to 13 hours, depending on factors such as the number of laborers, individual contributions, and environmental conditions such as land slope and field dispersion. Tractor-assisted spraying in Thailand requires shorter labor hours of 0.4–0.6 hours/ha. According to one surveyed farmer, drone-assisted spraying takes only 0.1 hours/ha.

In Vietnam, all surveyed farmers used a seed treatment containing thiamethoxam registered to control black cutworms, *Agrotis ipsilon* (Hufnagel) (Lepidoptera: Noctuidae), but not FAW. [**Supplementary Table S3** (117)]. Seeds were soaked in thiamethoxam for 20–30 minutes before sowing using pots. The cost ranged from $4.1–$8.6/ha, with negligible financial burden considering pot depreciation. The labor involved 2–3 individuals and took approximately 0.1–0.5 hours.

## 4 Discussion

### 4.1 Improvement of foliar application for chemical insecticides

Regarding insecticide foliar application, which is widely accepted by farmers, significant areas exists where research and dissemination remain essential. Reports of improper pesticide use from a human safety perspective exist in all MSEA countries, especially concerning fruit and vegetable production (118–120). For example, Laos, Cambodia, and Vietnam have reported numerous cases of pesticide poisoning among farmers (120). Good application techniques, use of personal protective equipment, and the time interval before re-entering the field after insecticide application should be further emphasized (121).

Additionally, from the perspective of improving maize productivity, room exists for enhancing foliar application methods in terms of cost and labor burden. Our research observed the use of knapsack sprayers over large areas, thus indicating the potential for labor-saving application technologies, such as drones, to be developed and disseminated combined with appropriate quantities of insecticide.

Meanwhile, during large-scale FAW outbreaks, following appropriate application frequencies and quantities is crucial. Clear and accessible information on these methods is necessary. Control instructions based on action thresholds from Thailand, Vietnam, and Myanmar were provided (**Supplementary Figure S1**), with Thailand notably issuing an easily understandable poster (27). Nevertheless, more precise information on how to manage large outbreaks, including insecticide application method and its rotation use, should be available.

### 4.2 Concerns about resistance evolution due to extensive use of EMB

Previous studies note substantial usage of chemical insecticide during sever FAW outbreaks. For example, estimated costs for insecticides and labor reached $193–$258/ha in Vietnam’s 2019 FAW outbreak (40), which significantly exceeded the $25/ha average reported by Vietnamese farmers in our survey. Similarly, maize farmers in Xundian County, northeastern Yunnan, China, increased pesticide applications from 2.1 at $81/ha in 2018 to 6.4 at $276/ha in 2020 per crop season (122).

Continuous use of a single active ingredient in chemical insecticides raises concerns about potential resistance evolution in FAW. Bioassay tests on samples collected from six maize-producing regions in Thailand revealed significantly higher lethal concentrations for 50% mortality of EMB, indoxacarb, and chlorfenapyr in 2021–2022 samples compared to those from 2019, thereby implying potential resistance evolution (123). The Arthropod Pesticide Resistance Database documents FAW’s field-evolved resistance to organophosphorus and pyrethroid pesticides, primarily in the Americas and China (124, 125), with emerging resistance evolution in FAW populations to new insecticides like spinosyns, diamides, triflumuron, and EMB, as well as to Cry1F *Bt* maize (125, 126).

### 4.3 Seed treatment as an alternative to EMB

Seed treatment would be a potential method to avoid the concentrative use of EMB in terms of efficacy, cost, and labor burdens. Numerous studies demonstrate the efficacy of seed treatment with diamides (e.g., chlorantraniliprole, cyantraniliprole) and neonicotinoids (e.g., thiamethoxam, clothianidin). Field tests in China have demonstrated significantly lower levels of plant damage by FAW compared to control groups when using combinations such as chlorantraniliprole + clothianidin and cyantraniliprole + thiamethoxam (127). In maize fields in India, the application of multiple seed treatment agents, including chlorantraniliprole, cyantraniliprole, and thiamethoxam, reduced foliar damage caused by FAW (128, 129). Additionally, in Zambia, seed treatments with cyantraniliprole + thiamethoxam resulted in decreased foliar damage by FAW, reduced field spread, increased yields, fewer foliar spray applications, and improved cost-benefit ratios (130–132).

Our survey of farmers in Vietnam confirms that seed treatment is cost-effective and labor-efficient. The Vietnamese government has approved a seed treatment containing cyantraniliprole + thiamethoxam for FAW control (117). The cost of the cyantraniliprole + thiamethoxam agent ($21–$27/ha) is significantly higher than that of thiamethoxam alone ($4–$9/ha), which our interviewed farmers used in Vietnam. Nevertheless, owing to its limited expenses relative to total production costs and ease of application, seed treatment remains a practical option.

Seed treatment efficacy typically lasts 14–21 days after sowing [**Supplementary Figure S1** (127, 128)]. Data from Phu Tho Province in Vietnam indicates a high rate of FAW infestation during this period (133). Understanding the general spread of FAW during the early stage and its impact on subsequent growing stages is crucial and this information could enhance the persuasiveness of recommending seed treatment.

### 4.4 Efficacy and cost-effectiveness of chemical insecticide alternatives

The strategies of MSEA governments aspiring toward IPM for FAW control are of significant importance in mitigating the risk of resistance evolution. In addition to chemical insecticides, we discuss the potential deployment and necessity for further verification of utilizing plant varieties, biological control, mass trapping, and local measures and agronomic practices for FAW control, which are often highlighted by MSEA governments.

#### 4.4.1 Host plant resistance: plant variety

Host plant resistance is considered a key component of IPM for controlling FAW as it can provide farmers an affordable option (134, 135). However, the information on non-GM FAW-resistant varieties provided by MSEA governments lacks specificity. Although Myanmar recommends the use of high-resistance varieties, the names of the recommended non-GM varieties are not specified [**Supplementary Table S1**; (78)]. Across Southeast Asia, research related to the screening of FAW-resistant varieties has been reported for Indonesia and the Philippines. However, these efforts have yet to lead to the recommendation of widely cultivated varieties (136–139).

By contrast, Vietnam, which is the only country in MSEA where GM maize cultivation is approved (103, 140), explicitly recommends specific GM maize varieties for use [**Supplementary Table S1** (95, 96)]. Trials have demonstrated the efficacy of GM maize in pest control (103, 141). In 2020, trials in Son La Province assessed GM maize’s resistance to FAW (141), and involved various *Bt* maize hybrids, such as DeKalb DK9955S and DK6919S, which introduced stacked events MON89034 (Cry1A.105 + Cry2Ab2) × NK603, and Syngenta NK7328Bt/GT, incorporating Bt11 × GA21 (103, 141). The results revealed dry weight yields averaging 7.6–7.8 t/ha, which significantly exceeds control maize varieties yielding 5.2–5.7 t/ha (141). A 2018–2019 nationwide field survey in Vietnam found GM varieties averaged 8.7 t/ha before de-husking and drying, compared to conventional varieties at 6.7 t/ha (103). Although GM seeds are more expensive at $131/ha compared to $102/ha for conventional varieties, their use resulted in a $330/ha increase in income, primarily due to higher yield (103). The same survey also established that GM seed adoption significantly reduced environmental impact by decreasing the area requiring insecticide applications and substantially lowering the time needed for field monitoring (103).

In the Philippines, GM maize, which was initially introduced on a commercial scale in 2003 to address Asian corn borer (*Ostrinia furnacalis* Guenée) (Lepidoptera: Crambidae) and weed issues, expanded to cover two-thirds of the total 1.42 million ha of yellow maize cultivation by 2019 (142). In addition to the positive evaluation from the economic analysis (142), the rapid expansion in the cultivation area alone implies the benefits that GM maize has provided to farmers.

#### 4.4.2 Biological control: release of natural enemies

A vast body of research on biological control exists, and use of such control for FAW control is anticipated (6, 143); however, widespread adoption has not been observed in MSEA. Thailand’s PPRDO distribute natural enemies for FAW control upon farmers’ requests, but domestic commercial production and market distribution remain unconfirmed. Our survey exhibits the limited use of natural enemies for FAW control in Thailand. Limited circulation and utilization may stem from uncertainties regarding the effectiveness of releasing natural enemies for biological control in outdoor environments (144). Although several biological control agents have been recommended for controlling FAW, the sources in **Supplementary Table S1** do not provide details on their specific application and effectiveness in MSEA. However, reports of experiments conducted in China and India provide insights into the performance of some natural enemies.

*Trichogramma chilonis* (Ishii) (Hymenoptera: Trichogrammatidae) significantly parasitized FAW eggs and reduced larval populations compared to the control. Trials involved releasing *T. chilonis* from cards with parasitized eggs: an estimated 22.5–30 adults/m^2^ twice in three maize fields ranging from 667 to 3,334 m^2^ (145), and 15 adults/m^2^ once for every 66.7 m^2^ (146). Additionally, Jin et al. (147) observed fewer damaged plants and FAW larvae when *Trichogramma* spp. parasitized eggs of rice moth, *Corcyra cephalonica* (Stainton) (Lepidoptera: Pyralidae), near FAW egg masses. In a Chinese experiment, *Telenomus remus* (Nixon) (Hymenoptera: Scelionidae) also exhibited high parasitism rates of egg masses within a 5 m radius (148). In Indian laboratory experiments, the predatory stink bug, *Eocanthecona furcellata* (Wolff) (Hemiptera: Pentatomidae), effectively preyed on small FAW instars, although concerns arose about its outdoor efficacy due to secondary parasitoids (149). Nonetheless, *E. furcellata* demonstrated a notable decrease in FAW when released at densities of 0.24 adults/m^2^ and 0.12 adults/m^2^ in a 50 m^2^ sweet corn field in China (150).

Assessing the feasibility of using natural enemies requires a thorough understanding of their propagation costs, which remain largely unknown in MSEA. In a pilot study conducted by the PPRDO, the production cost for an average of 3,631 stink bugs (*E. furcellata*) per month in a laboratory was $344 for the fixed costs (e.g., rearing containers), $442/month for variable costs (e.g., feed), and $3,428/month for labor costs (151). According to an interview with the PPRDO, the cost of a one-time release of 3,125 adults/ha (0.31 adults/m^2^) to manage FAW was estimated at $32/ha, which exceeds the median material cost of $9/ha for applying EMB. Although the relatively high variable and labor costs present challenges, scaling up production could reduce average costs, ultimately highlighting the need for further evaluation of its economic feasibility.

Owing to a certain effectiveness in FAW management and relatively low propagation costs, the potential for use of *Trichogramma* is worth considering (143, 152). As a particularly inexpensive example from Brazil, *T. pretiosum* (Riley) (Hymenoptera: Trichogrammatidae) and *T. galloi* (Zucchi) (Hymenoptera: Trichogrammatidae) are sold at a cost of $8–10/ha for the application of 100,000 parasitoids/ha, with an additional $2–3 for drone application (153). If similar costs are feasible in MSEA, their adoption could be viable at least from an economic perspective.

#### 4.4.3 Mass trapping with sweet-sour bait

The Vietnamese government considers mass trapping, particularly with sweet-sour bait, promising for FAW control [**Supplementary Table S1**; (154); Northern Plant Protection Center, 2019, unpublished data)]. An unpublished report from the Plant Protection and Cultivation Sub-Department in Hoa Bing Province, Vietnam, supports this finding. The report illustrates a decrease in FAW larval density in maize fields with 150 sweet-sour bait traps per ha compared to fields without traps (Northern Plant Protection Center, 2019, unpublished data).

However, as farmers often remove egg masses from fields with installed traps, this finding is debatable. Additionally, according to our estimation based on trial data from Gia Lai Province, installing 150 sweet-sour bait traps per ha costs significantly more at $142/ha than the median material cost of EMB at $9/ha (Central Plant Protection Center, 2020, unpublished data).

Given the limited global adoption and recommendation of mass trapping for FAW control (155, 156), further discussion on its efficacy and cost-effectiveness is necessary. Tay et al. (156) highlight challenges such as the high dispersal rate, multiple mating, and outbreak populations of FAW, which may hinder the effectiveness of pheromone-based mass trapping.

#### 4.4.4 Local measures and agronomic practices

Owing to potential variations influenced by factors such as farm scale, farming methods, surrounding vegetation, and the presence of natural enemies, the efficacy of local and agronomic control measures requires thorough validation. Instead of universally recommending these measures, determination of their effective conditions and establishment of a target range for implementation are vital.

Pest control methods recommended in Myanmar, Cambodia, and Vietnam, such as handpicking and whorl treatment—which involves spreading materials like sand, sawdust, and husk ash into leaf whorls—align with FAO guidelines for African farmers (85). However, their effectiveness is debated, and they impose significant labor burdens on larger-scale production (157–160).

Assessing the effectiveness of agronomic practices, particularly tillage and weed control, to prevent FAW infestation is challenging given the relationship with the conservation of indigenous natural enemies. Tillage recommendations vary—Thailand and Vietnam suggest deep tillage, while Myanmar advocates for conservative biological control without tillage. Conflicting information exists regarding the effectiveness of deep tillage, and its impact on FAW control remains uncertain (161–163). Similarly, the effects of no-tillage are not fully understood (162), although practices such as zero- and minimum-tillage, combined with manure or compost application, have been claimed to be effective in reducing FAW damage (161, 164).

Weeding is advised in Myanmar, Cambodia, and Vietnam (**Supplementary Table S1**). Frequent weeding may reduce FAW damage, particularly when potential hosts like graminaceous species dominate the weed population (161). However, weeds may serve as habitats for natural enemies and hinder the movement of small FAW larvae (163).

### 4.5 Enhancing the cost-effectiveness of chemical insecticide alternatives

Our research suggests that FAW management methods proposed as alternatives to chemical insecticides will not be adopted unless they are more efficacious and cost-effective than EMB foliar applications. Clarifying the cost-effectiveness of alternative technologies is fundamental to their improvement, and plant protection divisions/departments in MSEA have already conducted several studies. For example, Thailand’s PPRDO is evaluating both the effectiveness and costs of biological control (personal communication). In Vietnam, the Regional Plant Protection Centers established test sites in several regions immediately the invasion of FAW to conduct management trials. These trials included examining the field effectiveness and economic feasibility of various techniques, such as using resistant cultivars and mass trapping. Understanding the costs associated with such insecticide-alternative control methods and promoting research to reduce these costs represent critical first steps toward the dissemination of IPM-oriented technologies that national governments aim to promote.

## 5 Conclusion

This study synthesized the initial emergence of FAW infestations, government responses, and farmer reactions in MSEA. Furthermore, the feasibility of government-recommended measures was then examined in terms of efficacy and cost-efficiency.

From late 2018 to the rainy season of 2019, FAW infestations extensively emerged in maize fields in MSEA. Subsequently, MSEA governments promptly issued strategies and guidelines through plant protection divisions/departments, often in collaboration with international organizations, foreign governments, and private web portals.

Comparing the control recommendations of governments for FAW, the foliar application of EMB emerged as the most frequently mentioned method. Additionally, MSEA governments advocate for IPM-oriented approaches, which include application methods of chemical insecticides, use of host plant resistance, biological control, cultural and interference methods, and local measures aimed at reducing chemical usage.

Despite comprehensive recommendations from MSEA governments, our surveys reveal that maize farmers primarily rely on foliar treatment with EMB for FAW control. Insect control expenses, including input goods and application, generally represent less than 5% of total costs for surveyed farmers. The median costs for EMB and its application are as low as $9/ha and $5/ha, respectively, with a maximum of two applications per crop season.

Regarding the widely accepted practice of insecticide foliar application, further research and dissemination are essential in several areas. These include ensuring human safety through proper application methods, utilizing labor-saving application technologies, and providing clear and accessible information on application techniques.

While EMB is cost-effective, its widespread and concentrated use raises concerns about the evolution of resistance in FAW. Therefore, we explored the feasibility of several alternatives advocated by MSEA governments. Recent studies on the management suggest the efficacy of specific measures, including seed treatment with diamides and neonicotinoids, GM maize seeds, and biological control methods such as mass release of *Trichogramma* and predatory stink bugs. Seed treatment provides cost and labor benefits for preventing early-stage FAW infestation. Understanding the general spread of FAW during these stages and its impact on subsequent outbreaks would strengthen the recommendation. GM maize is effective and cost-efficient but permitted only in Vietnam within MSEA. Further validation of rearing costs for natural enemies may prove advantageous as preliminary estimates in Thailand suggest they could be relatively low. Meanwhile, the effectiveness of mass trapping FAW adults and local measures and agronomic practices for FAW control requires thorough validation, including their applicable conditions.

We conclude with the following points. Not all strategies recommended by the government or widely discussed are necessarily relevant at farm-level where issues arise. To propose more acceptable strategies at the farm-level, in addition to further verifying the effectiveness of FAW management techniques, understanding the actual responses of governments and farmers, with a particular emphasis on cost-efficiency, is crucial. Given the low cost of EMB foliar application, the techniques intended as its alternatives should be available at a significantly low cost, inclusive of the cost of labor.

## Data Availability

The original contributions presented in the study are included in the article/**Supplementary Material**. Further inquiries can be directed to the corresponding author.
