# Supplementary material for: Government and farmer responses to the fall armyworm outbreak in mainland Southeast Asia
Source: Front Insect Sci. 2025 Jan 22;4:1455585. doi: 10.3389/finsc.2024.1455585 (PMC11794807; doi:10.3389/finsc.2024.1455585)
Supplement: Supplementary file 1 [file Table1.docx]

Supplementary Material

# Supplementary Table S1 Measures to address FAW highlighted by MSEA nations, USAID and CIMMYT, and FAO

| **Method** | **Description** | **Country** | **Org.** | **Note** |
| --- | --- | --- | --- | --- |
| **Sampling and monitoring** | | | | |
| **Surveillance** |  | TC | U |  |
| **Insect traps** | Pheromone trap (M,UF); light trap (T); sweet-sour bait trap (C) | MTC | UF |  |
| **Scouting** | “W” scouting / “ladder” scouting (MT,U); scout 10–20 consecutive plants in 5 different locations (F) | MT | UF |  |
| **Chemical control** | | | | |
| **Recommendation of active ingredients** | Emamectin benzoate (MTLCV,UF); indoxacarb (MTLCV); chlorantraniliprole (MTLC,UF); flubendiamide (MTLC); spinetoram (TLV,F); methoxyfenozide + spinetoram (TL); chlorfenapyr (TL); lufenuron (TV); emamectin benzoate + λ-cyhalothrin (M); fipronil (C); spinosad (U); λ-cyhalothrin (U); thiamethoxam + λ-cyhalothrin (F) | MTLCV | UF |  |
| **Application** |  |  |  |  |
| **- Seed treatment** | Cyantraniliprole (TL); cyantraniliprole + thiamethoxam (V^*^,F) | TLV | UF |  |
| **- Foliar treatment** |  |  |  |  |
| - Spraying method | Spray target: Concentrate the spray on the top cone of the maize plant (e.g., using a conical nozzle) (T); spray the leaves evenly and spray on the corn stalks (V); foliar broadcast spray, spray into leaf whorls, or ear spray depends on maize growth stage and worm size (U); spray into leaf whorls (F) | TV | UF |  |
|  | Spray timing: spraying in the evening (T); spray in early morning or cool evening (V) | TV |  |  |
|  | Wear personal protective equipment | MT | U |  |
| **Insecticide resistance management (IRM)** | Alternate use of insecticides with different active ingredients to avoid resistance evolution of pests (V); switch chemical groups every 30 days (T) | MTLV | U |  |
| **Host plant resistance** | | | | |
| **Plant variety** |  |  | F |  |
| **- Native generic resistance** | Use high resistance variety (M) | M | U |  |
| **- Transgenic resistance** | Use GM maize (e.g., NK7328Bt/GT, NK4300Bt/GT, NK66Bt/GT, DK8639S, DK6919S (V))^**^ | V | U |  |
| **Biological control** | | | | |
| **Insect natural enemies** |  |  |  |  |
| **- Egg parasitoids** | *Trichogramma* spp. (MTV,UF); *Telenomus* spp. (M,U) | MTV | UF |  |
| **- Larval parasitoids** | *Bracon hebetor* (F) |  | F |  |
| **- Predators** | Earwigs (V) or brown earwig (*Proreus simulans* (T)); predatory stink bugs (V) (*Eocanthecona furcellata* (T)); assassin bugs (*Sycanus* spp. (T)); ladybug (V) | TCV |  |  |
| **Pathogens** |  |  |  |  |
| **- Bacteria** | *Bacillus thuringiensis var. aizawai* or *kurstaki* | MTLV | U |  |
| **- Fungi** | *Metarhizium* (V) (*M. rileyi*, *M. anisopliace* (F)); *Beauveria* (V) | V | UF |  |
| **- Viruses** | *Nucleopolyhedrovirus* / SfNPV | V | UF |  |
| **- Entomopathogenic nematodes** | *Steinernematidae*, *Heterorhabditidae* (U) |  | U |  |
| **Conservative biological control** | No tillage, retain crop residues, and perform crop rotation to encourage beneficial insects (M); restrict use of chemical pesticides (V) | MTV | U |  |
| **Cultural and interference methods** | | | | |
| **Agronomic practices** |  |  |  |  |
| **- Land preparation** |  |  |  |  |
| - Ploughing | Plough and dry the soil to remove worms and pupae for severe outbreak (T) | TV |  | D (1,2,3) |
| - Using cover crops | Use of cover crops such as mucuna and lablab (M) | M |  |  |
| - Digging trenches | Dig a trench along the field and sprinkle ash or spray diesel along the trench to prevent FAW (C) | C |  | D (4,5) |
| **- Sowing** |  |  |  |  |
| - Short duration variety | Use short-duration maize varieties (F) |  | F |  |
| - Timely sowing | Sow maize as soon as the rain falls in early monsoon or water recedes from submerged areas (M) | M | UF | N (3) |
|  | Sow maize at the same time (F) |  | F |  |
| - Intercropping | Intercrop cassava, cowpeas, pigeon peas, sweet potato, sweet pea, beans, pumpkin, or other fodder crops, row by row (M); intercropping with cowpea, pigeon pea, and soybean can reduce oviposition of FAW on maize according to evidence from Africa and the Americas (U) | M | UF | N (3,6) |
| - Natural enemy pulling plants | Grow sunflowers, beans, and so on, in free spaces and at the boundary-periphery of maize fields to enhance beneficial insects (M) | M | UF | N (3) |
|  | Grow large trees such as Tamarisk or neem trees, or suburbs to increase birds (M) | M | U |  |
| - FAW pulling plants / objects | Plant elephant grass and sticky corn earlier to attract adults to lay eggs (V); create habitats of worms with objects such as fresh twigs or banana leaves to cast shade several places in the field (C) | CV |  |  |
| - Push-pull system | Intercrop maize with pest-repellent crops and plant other crops around the field (M); intercrop *Desmodium*. Plant Napier grass (*Pennisetum purpureum*) or *Brachiaria* spp. along the field boundary to attract FAW (M) | M | U | N (3,6) |
| - Encouraging refugia | Encourage refugia, parts of fields where crops are not sprayed with pesticides and are planted adjacent to transgenic crops, or natural areas within or adjacent to agricultural fields (U) |  | U |  |
| - Crop rotation | Perform crop rotation, particularly with wet-field rice cultivation (V) | V |  |  |
| **- Cropping** |  |  |  |  |
| - Nutrient management | Apply enough fertilizer (M) | M | UF |  |
| - Weeding | Eliminate grassy weed (M) | MCV | F | N (3) |
| **Semiochemicals** |  |  |  |  |
| **- Mass trapping to catch adult** | Sweet-sour bait trap (V); sweet bait trap (L); pheromone trap (LV,F) | LV | F | N (7) |
| **Physical methods** |  |  |  |  |
| **- Mass trapping to catch adult** | Light trap (LV) | LV |  |  |
| **Other methods** | | | | |
| **Botanical insecticides** | Azadirachtin (UF) |  | UF |  |
| **Local measures** |  |  |  |  |
| **- Hand picking** | Twice a week when FAW oviposition is heavy, and after that at a weekly or fortnightly interval (M) | MTV | F | N (8) |
| **- Whorl treatment** | Put ash, burned rice husk, sand, saw-dust, or dust into the whorl of maize leaf to kill larvae (M); pour water into the leaf whorl (M); pour kitchen ash or diluted soapy water into the leaf whorl (V) | MV |  | D (1,2,8) |
| **- Home-made insecticides** | Botanical insecticides: Spray solution of de-husked seed or leaves of neem (*Azadirachta indica*) (M); 6–8 kg of neem kernels/ha of maize (M); spray pyrethrum leaf solution (M); 5% neem seed kernel extract or 4% neem soap at 2mL/L water (F) | M | F |  |
|  | Spray lime/salt/oil/soap solution (M); spray diluted water composed of dead larvae naturally infected with entomopathogens (M); for waterlogged fields or near water sources, remove FAW and sprinkle sand mixed with diesel (C) | MC |  |  |
| **- Attracting natural enemies** | Spray sugar solution, molasses, and so on, in the field to attract natural enemies (M) | M |  | N (1,2,8) |

Abbreviations of countries: “M” = Myanmar, “T” = Thailand, “L” = Laos, “C” = Cambodia, “V” = Vietnam. Abbreviations of organizations (“Org.”): “U” = USAID and CIMMYT, “F” = FAO. Abbreviations in the Note column: “D” = Doubtful effectiveness, “N” = Further verification needed, according to prior studies. Sources for the Note column are in brackets. ^*^Source is the Ministry of Agriculture and Rural Development, Vietnam (9). ^**^GM varieties NK6101Bt/GT and DK99558S are also recommended in Vietnam by the letter No. 218/QD-BNN-BVT (9), although their circulation or FAW-resistance could not be confirmed. Instead, FAW-resistant varieties NK67Bt/GT and DK9955S have been identified (10, 11). Mating disruption by synthetic pheromone and self-limiting insect technology through inundative release of the self-limiting males to mate with wild-type females (12), have been omitted.

Sources: Myanmar (2, 6), Thailand (13–16), Vietnam (9, 17, 18), Laos (19), Cambodia (20–22), USAID and CIMMYT (12), and FAO (7).

# Supplementary Figure S1 Major control techniques for FAW and their action thresholds (AT) according to maize growth stage

Abbreviations for the maize growth stage: “VE” = emergence, “V(n)” = n leaves with visible leaf collars, “VT” = tasseling, “R1” = blister, “R5” = dent, “R6” = maturity. “Key farmers” = village-level progressive farmers, according to McGrath et al. (23), one of the sources of Myanmar’s strategy. “% dmg.” = the percentage of damaged plants out of total plants. “1st catch” = the first catch of 3 moths per trap. “bot. insecticides” = botanical insecticides. “SFW” = small, fresh windowpanes. “IW” = infested whorls. “IP” = infested plants.

Information from various sources was synthesized to clarify the overall picture, although there were inconsistencies, such as in FAO and the Plant Protection Division (6) for Myanmar and the Department of Agriculture (24) and sources for this table in Thailand.

For Thailand, chemical insecticide application amounts vary: “Chem. (a)” = 187.5–250 L water/ha for 1–2-week-old maize, “Chem. (b)” = 250–312.5 L water/ha for 3–4-week-old maize, “Chem. (c)” = 375–500 L water/ha for after 4-week-old to near harvest maize (25). Regarding the foliar application of chemical insecticides, it is advised to continue until VT, based on the statement, “spraying after the emergence of the ear from leaf sheath will not be effective” (16). The Department of Agriculture Thailand (24) provides more specific timing instructions, suggesting that seed treatment remains effective 21 days after sowing, chemical insecticides should be applied 1–2 times between 22–45 days after sowing, and microbial insecticides are recommended from 46 days after sowing until harvest. Monitoring is advised on the 21st–22nd day and the 45th–46th day after sowing.

Sources: Days after emergence and the vulnerability of maize to FAW (26), Myanmar (2, 6), Thailand (13–16), Vietnam (9, 17, 18), USAID (USAID and CIMMYT; 12), and FAO (7).

# Supplementary Table S2 Revenue and cost of maize production based on surveys and previous studies in MSEA

| **Country & season** | **Year** | **No.** | **Total area (ha)** | **Maize production** | | | **Profit, revenue, and cost (US$/ha)** | | | | | | | | | | | | | | **Obs. & source** |
| --- | --- | --- | --- | --- | --- | --- | --- | --- | --- | --- | --- | --- | --- | --- | --- | --- | --- | --- | --- | --- | --- |
|  |  |  |  | **Area (ha)** | **Yield (t/ha)** | **Std. yield (t/ha)** | **Profit** | **Revenue** | **Cost** | | | | | | | | | | | |  |
|  |  |  |  |  |  |  |  |  | **Total** | **Explicit cost** | | | | | | | **Implicit cost** | | | **Opr. cost** |  |
|  |  |  |  |  |  |  |  |  |  | **Total** | **Input goods** | **Outso-urcing** | **Fuel** | **Land rent** | **Irriga-tion** | **Other** | **Total** | **Depr.** | **Fam. labor** |  |  |
| **Myanmar** | | | | | | | | | | | | | | | | | | | | | |
| Wet | 2022 | 1 | 4.5 | 2.8 | 3.7 | 3.7 | 691 | 1,185 | 494 | 463 | 181 | 282 | 0 | 0 | 0 | 0 | 30 | 1 | 30 | 312 | Auth. |
|  | 2022 | 2 | 5.1 | 2.8 | 2.8 | 2.8 | 227 | 858 | 630 | 603 | 279 | 179 | 22 | 123 | 0 | 0 | 28 | 12 | 16 | 229 | Auth. |
|  | 2022 | 3 | 10.5 | 6.5 | 4.2 | 4.1 | 1,016 | 1,356 | 340 | 288 | 193 | 89 | 6 | 0 | 0 | 0 | 52 | 5 | 47 | 147 | Auth. |
|  | 2017 | ^*^ | N/A | 1.9 | 3.5 | N/A | 181 | 592 | 412 | 268 | 131 | 133 | N/A | N/A | N/A | 5 | 143 | N/A | 143 | — | 828 (27) |
| N/A | 2019/20 | ^*^ | N/A | N/A | N/A | N/A | 305 | 804 | 499 | 186 | 186 | N/A | N/A | N/A | N/A | N/A | N/A | N/A | N/A | 314 | N/A (28) |
| **Thailand** | | | | | | | | | | | | | | | | | | | | | |
| Wet | 2022 | 1 | 3.1 | 2.7 | 7.2 | 5.8 | 830 | 1,433 | 604 | 604 | 275 | 329 | 0 | 0 | 0 | 0 | 0 | 0 | 0 | 329 | Auth. |
|  | 2022 | 2 | 4.8 | 4.8 | 6.3 | 5.1 | 576 | 1,250 | 674 | 459 | 398 | 0 | 60 | 0 | 0 | 0 | 215 | 202 | 14 | 276 | Auth. |
|  | 2022 | 3 | 8.0 | 8.0 | 7.5 | 5.7 | 1,154 | 1,685 | 532 | 483 | 240 | 136 | 107 | 0 | 0 | 0 | 49 | 41 | 8 | 292 | Auth. |
|  | 2022 | 4 | 16.5 | 16.0 | 9.2 | 7.4 | 1,516 | 2,152 | 636 | 608 | 273 | 163 | 171 | 0 | 0 | 0 | 28 | 9 | 19 | 362 | Auth. |
|  | 2020 | ^*^ | N/A | N/A | 5.5 | N/A | N/A | 1,181 | N/A | 363 | 291 | N/A | 73 | N/A | 0 | N/A | N/A | N/A | N/A | — | 131 (29) |
| Dry | 2022 | 2 | 4.8 | 1.6 | 6.3 | 5.1 | 576 | 1,250 | 674 | 459 | 396 | 0 | 56 | 0 | 6 | 0 | 216 | 202 | 14 | 272 | Auth. |
|  | 2020 | ^*^ | N/A | N/A | 7.6 | N/A | N/A | 1,785 | N/A | 424 | 306 | N/A | 74 | N/A | 44 | N/A | N/A | N/A | N/A | — | 131 (29) |
| N/A | 2020 | ^*^ | 6.4 | 5.6 | N/A | N/A | N/A | N/A | 739 | 348 | 301 | N/A | N/A | 47 | N/A | N/A | 169 | N/A | 169 | 221 | 127 (Auth.) |
|  | 2015/16 | ^*^ | N/A | 6.1 | 5.4 | N/A | N/A | N/A | 602 | 298 | 298 | N/A | N/A | N/A | N/A | N/A | N/A | N/A | N/A | 304 | 404 (30) |
|  | 2014/15 | ^*^ | N/A | 2.3 | 7.2 | N/A | N/A | N/A | 901 | 701 | 701 | N/A | N/A | N/A | N/A | N/A | N/A | N/A | N/A | 200 | 52 (31) |
| **Laos** | | | | | | | | | | | | | | | | | | | | | |
| Wet | 2014/15 | ^*^ | N/A | 0.9 | N/A | 3.5 | N/A | N/A | N/A | 172 | 172 | N/A | N/A | N/A | N/A | N/A | N/A | N/A | N/A | 107 | 26 (32) |
| N/A | 2018 | ^*^ | N/A | N/A | 4.9 | N/A | 398 | 653 | 255 | 153 | 153 | N/A | N/A | N/A | N/A | N/A | N/A | N/A | N/A | 102 | 18 (33) |
| **Cambodia** | | | | | | | | | | | | | | | | | | | | | |
| Wet | 2022 | 1 | 3.0 | 3.0 | 5.0 | 3.8 | 60 | 1,028 | 968 | 968 | 621 | 322 | 0 | 24 | 0 | 0 | 0 | 0 | 0 | 322 | Auth. |
|  | 2022 | 2 | 8.0 | 7.0 | 5.0 | 3.8 | 181 | 979 | 798 | 680 | 380 | 149 | 120 | 31 | 0 | 0 | 118 | 104 | 15 | 387 | Auth. |
| Dry | 2022 | 1 | 3.0 | 3.0 | 7.0 | 5.3 | 809 | 1,832 | 1,023 | 1,023 | 621 | 322 | 0 | 24 | 55 | 0 | 0 | 0 | 0 | 322 | Auth. |
|  | 2022 | 2 | 8.0 | 8.0 | 7.0 | 5.3 | 938 | 1,713 | 774 | 655 | 365 | 146 | 113 | 31 | 0 | 0 | 119 | 104 | 15 | 378 | Auth. |
| N/A | 2013 | ^*^ | N/A | 1.4 | 7.5 | N/A | 705 | 1,943 | 1,238 | 908 | 581 | N/A | 145 | 133 | 30 | 19 | N/A | N/A | N/A | 330 | 96 (34) |
| **Vietnam** | | | | | | | | | | | | | | | | | | | | | |
| Wet | 2022 | 1 | 2.0 | 2.0 | 12.5 | 10.5 | 1,780 | 3,046 | 1,266 | 1,119 | 760 | 342 | 17 | 0 | 0 | 0 | 148 | 6 | 141 | 507 | Auth. |
|  | 2022 | 2 | 3.2 | 2.2 | 3.6 | 3.3 | −932 | 933 | 1,865 | 906 | 712 | 155 | 39 | 0 | 0 | 0 | 959 | 4 | 955 | 1,153 | Auth. |
|  | 2022 | 3 | 4.0 | 3.0 | 10.0 | 8.7 | 327 | 2,865 | 2,538 | 1,158 | 1,028 | 81 | 49 | 0 | 0 | 0 | 1,380 | 288 | 1,091 | 1,510 | Auth. |
|  | 2022 | 4 | 7.0 | 7.0 | 7.9 | 7.3 | 1,078 | 2,184 | 1,106 | 974 | 564 | 380 | 25 | 5 | 0 | 0 | 132 | 8 | 124 | 537 | Auth. |
|  | 2022 | 5 | 8.0 | 7.0 | 9.3 | 8.4 | 1,157 | 2,700 | 1,543 | 1,325 | 870 | 191 | 24 | 241 | 0 | 0 | 218 | 6 | 212 | 432 | Auth. |
| Dry | 2022 | 1 | 2.0 | 2.0 | 10.0 | 8.7 | 1,255 | 2,565 | 1,310 | 1,163 | 688 | 453 | 21 | 0 | 0 | 0 | 148 | 6 | 141 | 622 | Auth. |
| Both | 2015/16 | ^*^ | N/A | 0.8 | N/A | N/A | 585 | 1,376 | 795 | 565 | 452 | N/A | N/A | 8 | 12 | 94 | 18 | 18 | N/A | 333 | 222 (35) |

“No.” = number of surveyed farmers in each country. ^*^Data estimated from the previous study. “Total area” = total agricultural area. “Area” = cultivated area. “Std. yield” = standardized yield with 14% moisture content. “Profit” = Revenue − Total cost. “Depr.” = depreciation cost of fixed assets, including machines and equipment. “Fam. labor” = opportunity cost for family laborers estimated from person-hours multiplied by the minimum wage in each country. “Opr. cost” = operation cost, which includes some factors in explicit and implicit costs. For the 2022 value, operation cost is the sum of the fee for outsourcing and fuel, depreciation, and family labor costs. In past studies, operation costs may include costs for hired and family labor and other costs such as fuel, equipment, machines, and depreciation. “Obs.” = number of observations in past studies. Sources are in brackets. “Auth.” = authors’ surveys in 2022 and 2023. “N/A” = not available.

The values from past studies were converted into real values in each country’s national currency unit for 2022 using CPI (36) and the exchange rates for each year. Subsequently, the values in national currency unit were converted into US$ with the following exchange rates (national currency unit/US$) in 2022: Myanmar = 1,928, Thailand = 35.01, Laos = 14,370, Cambodia = 4,087, Vietnam = 23,388 (37).

# Supplementary Table S3 Responses and associated costs of farmers infested by FAW, wet season in 2022

| **Country** | **No.** | **Maize area (ha)** | **Days after sowing** | **Infested area (%)** | **Insecticide** | | | | | **Application** | | | | | | | | |
| --- | --- | --- | --- | --- | --- | --- | --- | --- | --- | --- | --- | --- | --- | --- | --- | --- | --- | --- |
|  |  |  |  |  | **Purpose** | **Active ingredient** | **Cost (US$/**  **ha)** | **Qty. (volume/**  **unit)** | **Price (US$/**  **volume)** | **Equipment /**  **machine** | **Cost (US$/ha)** | | | | **Price (US$/**  **unit)** | **Workload** | | |
|  |  |  |  |  |  |  |  |  |  |  | **Total** | **Outsourcing** | **Fuel** | **Depr.** |  | **Wkng. hr. (hr/ha)** | **N. of laborers (pers.)** | |
|  |  |  |  |  |  |  |  |  |  |  |  |  |  |  |  |  | **Total** | **Family** |
| **Foliar treatment** | | | | | | | | | | | | | | | | | | |
| Myanmar | 1 | 2.8 | 30 | 80 | Ext., 1^st^ | EMB 5% (G) | 1.3 | 0.35 | 3.6 | Knp. manual | 5.5 | 5.1 | 0.0 | 0.3 | 15.5 | 2.1 | 6 | 2 |
|  |  |  | Ditto | Ditto | Ext., 1^st^ | EMB 2% +  IDX 16% (L) | 8.8 | 0.71 | 12.4 | Ditto | Ditto | Ditto | Ditto | Ditto | Ditto | Ditto | Ditto | Ditto |
|  | 2 | 2.8 | 30 | 10 | Prv., 1^st^ | LCY 5% (L) | 3.7 | 0.35 | 10.6 | Knp. manual | 3.5 | 2.9 | 0.0 | 0.6 | 15.5 | 2.8 | 3 | 1 |
| Thailand | 1 | 2.7 | 30 | 20 | Ext., 1^st^ | EMB 5% WG | 8.9 | 0.63 | 14.3 | Tractor | 12.5 | 12.5 | 0.0 | 0.0 | — | N/A | 1 | 0 |
|  | 2 | 4.8 | 40 | 25 | Ext., 1^st^ | EMB 5% WG | 8.9 | 0.63 | 14.3 | Tractor | 4.6 | 0.0 | 1.2 | 3.3 | 5,712.7 | 0.6 | 1 | 1 |
|  |  |  | 40 | 25 | Ext., 2^nd^ | EMB 5% WG | 8.9 | 0.63 | 14.3 | Tractor | 4.6 | 0.0 | 1.2 | 3.3 | 5,712.7 | 0.6 | 1 | 1 |
|  | 3 | 8.0 | 40 | 100 | Ext., 1^st^ | EMB 5% WG | 8.9 | 0.63 | 14.3 | Tractor | 14.3 | 14.3 | 0.0 | 0.0 | — | N/A | 1 | 0 |
|  | 4 | 16.0 | 20 | 30 | Ext., 1^st^ | EMB 5% WG | 8.9 | 0.63 | 14.3 | Tractor | 7.8 | 0.0 | 5.5 | 2.3 | 5,712.7 | 0.4 | 1 | 1 |
|  |  |  | 70 | 20 | Ext., 2^nd^ | EMB 5% WG | N/A | N/A | N/A | Drone | 18.8 | 18.8 | 0.0 | 0.0 | — | 0.1 | 2 | 0 |
| Cambodia | 2 | 7.0 | 30 | 0 | Prv., 1^st^ | EMB 4% +  PMT 50% (L) | 7.9 | 0.79 | 10.0 | Knp. power | 4.0 | 2.9 | 0.9 | 0.3 | 94.9 | 2.0 | 2 | 1 |
|  |  |  | 50 | 0 | Prv., 2^nd^ | EMB 4% +  PMT 50% (L) | 7.9 | 0.79 | 10.0 | Knp. power | 4.0 | 2.9 | 0.9 | 0.3 | 94.9 | 2.0 | 2 | 1 |
| Vietnam | 1 | 2.0 | 30 | 40 | Ext., 1^st^ | EMB 6% WG^a^ | 17.1 | 0.60 | 28.5 | Knp. power | 12.4 | 8.6 | 3.2 | 0.6 | 256.5 | 4.0 | 4 | 2 |
|  |  |  | 40 | 30 | Ext., 2^nd^ | EMB 6% WG | 17.1 | 0.60 | 28.5 | Knp. power | 22.6 | 17.1 | 4.3 | 1.2 | 256.5 | 8.0 | 4 | 2 |
|  | 2 | 2.2 | 60 | 40 | Ext., 1^st^ | EMB 5% WG^a^ | 11.7 | 0.41 | 28.5 | Knp. power | 3.1 | 0.0 | 2.0 | 1.1 | 213.8 | 12.7 | 5 | 5 |
|  |  |  | 60 | 30 | Ext., 2^nd^ | EMB 5% WG | 11.7 | 0.41 | 28.5 | Knp. power | 3.6 | 0.0 | 2.7 | 0.9 | 213.8 | 10.9 | 5 | 5 |
|  | 3 | 3.0 | 40 | 40 | Ext., 1^st^ | EMB 5% WG | 4.3 | 0.30 | 14.3 | Knp. power | 3.7 | 0.0 | 3.2 | 0.5 | 128.3 | 13.3 | 4 | 4 |
|  |  |  | 40 | 30 | Ext., 2^nd^ | EMB 5% WG | 4.3 | 0.30 | 14.3 | Knp. power | 3.5 | 0.0 | 3.2 | 0.3 | 128.3 | 8.0 | 5 | 5 |
|  | 4 | 7.0 | 30 | 40 | Ext., 1^st^ | EMB 5% WG^a^ | 9.8 | 0.86 | 11.4 | Stationary | 47.8 | 38.5 | 7.3 | 2.0 | 427.6 | 8.0 | 7 | 2 |
|  |  |  | 30 | 30 | Ext., 2^nd^ | EMB 5% WG^b^ | 1.7 | 0.15 | 11.4 | Stationary | 0.8 | 0.0 | N/A | 0.8 | 427.6 | N/A | 2 | 2 |
|  | 5 | 7.0 | 60 | 30 | Prv., 1^st^ | EMB 5% WG^a^ | 24.4 | 0.86 | 28.5 | Stationary | 4.7 | 0.0 | 3.2 | 1.5 | 470.3 | 4.0 | 6 | 6 |
| **Seed treatment** | | | | | | | | | | | | | | | | | | |
| Vietnam | 1 | 2.0 | — | — | — | TMT 35% FS | 7.7 | 3.0 | 128.3 | Pot | 0.0 | 0.0 | 0.0 | 0.0 | N/A | 0.5 | 2 | 2 |
|  | 2 | 2.2 | — | — | — | TMT 35% FS | 5.2 | 2.0 | 128.3 | Pot | 0.0 | 0.0 | 0.0 | 0.0 | N/A | 0.5 | 2 | 2 |
|  | 3 | 3.0 | — | — | — | TMT 35% FS | 8.6 | 3.0 | 142.5 | Pot | 0.0 | 0.0 | 0.0 | 0.0 | N/A | 0.3 | 3 | 3 |
|  | 4 | 7.0 | — | — | — | TMT 35% FS | 8.6 | 3.0 | 142.5 | Pot | 0.0 | 0.0 | 0.0 | 0.0 | N/A | 0.1 | 2 | 2 |
|  | 5 | 7.0 | — | — | — | TMT 35% FS | 4.1 | 1.6 | 128.3 | Pot | 0.0 | 0.0 | 0.0 | 0.0 | N/A | 0.3 | 2 | 2 |

“Days after sowing” = The number of days after sowing is estimated based on a calendar divided into 10-day intervals, with recorded dates for sowing and pesticide application. Thus, it may vary by up to 10 days in either direction.

“Purpose”: “Ext.” = extraction of worms, “Prv.” = prevention of worms. 1st and 2nd denote 1st time and 2nd time application, respectively. “Active ingredient”: “EMB” = emamectin benzoate, “PMT” = permethrin, “IDX” = indoxacarb, “LCY” = λ-cyhalothrin, “TMT” = thiamethoxam. Dosage form: “WG” = water dispersible granules, “FS” = flowable concentrate for seed treatment. The detailed dosage form is unclear: “(G)” = granule, “(L)” = liquid. ^a^Mixed EB with herbicide, such as atrazine 50% + mesotrione 5% SC (suspension concentrate) by the farmer and sprayed together. ^b^Applied to a part of the total area. “Qty.” = quantity. The unit of quantity for WG and (G) = kg/ha, for (L) = L/ha, and for TMT 35% FS used in seed treatment = mL/kg seed.

“Equipment/machine”: “Knp. Manual” = knapsack manual sprayer, “Knp. Power” = knapsack power sprayer, “Stationary” = stationary engine sprayer. “Depr.” = depreciation of equipment and machines. “Price” = unit price of equipment or machine. “Workload”: “Wkg. hr.”" = working hour per ha. “N. of laborers” = number of laborers (persons). “Family” = family laborers. “N/A” = not available.

Source: Authors’ surveys in 2022 and 2023.

# References

1. Thierfelder C, Niassy S, Midega C, Subramanian S, Van Den Berg J, Prasanna BM, et al. “Low-cost agronomic practices and landscape management approaches to control FAW”. In: Prasanna BM, Heusing JE, Eddy R, Peschke, editors. Fall Armyworm in Africa: A Guide for Integrated Pest Management (first edition). Mexico City: CIMMYT (2018) 89-95.

2. Plant Protection Division, Ministry of Agriculture, Livestock and Irrigation, Myanmar. Spread Situation and Methods of Prevention and Control of Fall Armyworm in Myanmar. Yangon: Plant Protection Division (2019) 1-54. (in Burmese).

3. Krupnik, TJ. “Agroecological management of fall armyworm in Asia”. In: Prassana BM, Huesing JE, Peschke VM, Eddy R, editors. Fall Armyworm in Asia: A Guide for Integrated Pest Management. Mexico City: CIMMYT (2021) 138-153.

4. Department of Agriculture, Ministry of Agriculture, Livestock and Irrigation, Myanmar. FAW prevention and control methods. Green Way. 2019 Jan 14. (2019). <https://greenwaymyanmar.com/posts/FAW_Armyworm_control_DoA> [accessed June 18, 2024] (in Burmese).

5. Department of Agriculture, Ministry of Agriculture, Livestock and Irrigation, Myanmar. FAW prevention and control methods. Green Way. 2019 Jun 5. (2019). <https://greenwaymyanmar.com/posts/protection_of_faw_army_worm> [accessed June 18, 2024] (in Burmese).

6. Food and Agriculture Organization of the United Nations and Plant Protection Division, Department of Agriculture, Ministry of Agriculture, Livestock, and Irrigation, Myanmar. Manual on Integrated Fall Armyworm Management. Yangon: FAO. (2020). doi: 10.4060/ca9688en

7. Food and Agriculture Organization of the United Nations. Technical Guidance on Fall Armyworm: Coordinated Surveillance and an Early Warning System for the Sustainable Management of Transboundary Pests, with Special Reference to Fall Armyworm (*Spodoptera frugiperda* [J.E. Smith]) in South and Southeast Asia. Bangkok: FAO. (2022). doi: 10.4060/cc0227en

8. McGrath DM, Jepson PC, Huesing JE, Defrancesco J, Deshmukh SS, Peschke VM, et al. “Pesticide application, safety, and selection criteria for fall armyworm control”. In: Prassana BM, Huesing JE, Peschke VM, Eddy R, editors. Fall Armyworm in Asia: A Guide for Integrated Pest Management. Mexico City: CIMMYT (2021) 58-98.

9. Ministry of Agriculture and Rural Development, Vietnam. Technical procedures for prevention and control of fall armyworm (*Spodoptera frugiperda*). 6/TTDVNN. Accessed through Ia H'Drai District People’s Committee. (2020). <http://www.iahdrai.kontum.gov.vn/VBDetail.aspx?idVB=2145> [accessed June 18, 2024] (in Vietnamese).

10. Brookes G, Dinh TX. The impact of using genetically modified (GM) corn/maize in Vietnam: results of the first farm-level survey. *GM Crops Food*. (2021) 12:71-83. doi: 10.1080/21645698.2020.1816800

11. Nguyen TD, Dao HTL, Pham XT. Testing of genetically modified maize varieties with resistant ability to fall armyworm in Son La province. *J Vietnam Agri Sci Tech*. (2022) 3:92-98 (in Vietnamese).

12. Prasanna BM, Huesing JE, Peschke VM, Eddy R. Fall Armyworm in Asia: A Guide for Integrated Pest Management. Mexico City: CIMMYT. (2021).

13. Plant Protection Research and Development Office, Department of Agriculture, Ministry of Agriculture and Cooperatives, Thailand. Prevention and Elimination Fall Armyworm *Spodoptera frugiperda* (J.E. Smith). (2019). <https://www.doa.go.th/plprotect/?page_id=3090> [accessed June 18, 2024] (in Thai).

14. Plant Protection Promotion and Soil-Fertilizer Management Division, Department of Agricultural Extension, Thailand. Maize pest management recommendations (7 pest types), pest management decision guide (PMDG). (2021). <http://www.ppsf.doae.go.th/wordpress/?p=10854> [accessed June 18, 2024] (in Thai).

15. Plant Protection Promotion and Soil-Fertilizer Management Division, Department of Agricultural Extension, Thailand. Warning of fall armyworm infestation. Weekly Pest Outbreak Warning News, 9. 2022 Jul 13. (2022) <http://www.ppsf.doae.go.th/wordpress/?p=12924> [accessed June 18, 2024] (in Thai).

16. Nakhon Sawan Field Crops Research Center, Thailand. Fall armyworm. (2023). <https://www.doa.go.th/fc/nakhonsawan/?p=1332> [accessed June 18, 2024] (in Thai).

17. Plant Protection Department, Vietnam. List of Active Ingredients for Fall Armyworm Prevention, issued together with Official Dispatch No. 3749/BVTV-QLT. Hanoi: Plant Protection Department. (2019). (in Vietnamese).

18. National Agricultural Extension Center, Vietnam. Technical Guidance on Fall Armyworm Prevention and Control, 2021 Reprint. (2021). <https://khuyennongvn.gov.vn/thu-vien-khuyen-nong/thu-vien-sach-kn/huong-dan-ky-thuat-phong-chong-sau-keo-mua-thu-tai-ban-nam-2021-21351.html> [accessed June 18, 2024] (in Vietnamese).

19. Plant Protection Center, Department of Agriculture, Lao PDR. Fall armyworm, *Spodoptera frugiperda* (J.E. Smith). (2019). https://lao44.org/content/3189/ບົ້ງຝູງສາລີ [accessed June 18, 2024] (in Lao).

20. Plant Protection, Sanitary, and Phytosanitary Department, General Directorate of Agriculture, Ministry of Agriculture, Forestry and Fisheries, Cambodia. Control measures of fall armyworm. Accessed through Kampong Chhnang Provincial Department of Agriculture, Forestry, and Fisheries. 2019 Jul 4. (2019). <https://kampongchhnang.maff.gov.kh/post/5d1db2ef9c25c> [accessed June 18, 2024] (in Khmer).

21. Tboung Khmum Provincial Department of Agriculture, Forestry, and Fisheries. Fall armyworm. 2020 Jun 12. (2020). <https://www.facebook.com/agronomy.tboungkhmum.5> [accessed June 18, 2024] (in Khmer).

22. Pailin Provincial Department of Agriculture, Forestry and Fisheries. Mr. Say Sophat, director of the department, intervened and introduced the technical rules to get rid of fall armyworms. 2021 May 6. (2021). <https://pailin.maff.gov.kh/post/6092b5bd7b5b9> [accessed June 18, 2024] (in Khmer).

23. McGrath DM, Huesing JE, Beiriger R, Nuessly G, Tepa-Yotto TG, Hodson D, et al. “Monitoring, surveillance, and scouting for fall armyworm”. In: Prasanna BM, Heusing JE, Eddy R, Peschke, editors. Fall Armyworm in Africa: A Guide for Integrated Pest Management (first edition). Mexico City: CIMMYT (2018) 11-28.

24. Department of Agriculture, Ministry of Agriculture and Cooperatives, Thailand. Prevention and elimination of the fall armyworm in various types of corn poster. (2019). <https://www.doa.go.th/plprotect/?p=3028> [accessed June 18, 2024] (in Thai).

25. Sukonthaphirom S, Phatthalung N, Boonwatho P, Phopoonsak S, Srichandra S. Advice on Safety Pest Prevention and Control: From Research in 2021. Academic Document. Bangkok: PPRDO. (2021). (in Thai).

26. McGrath DM, Huesing JE, Jepson PC, Peschke VM, Prasanna BM, Krupnik TJ. “Fall armyworm scouting, action thresholds, and monitoring”. In: Prassana BM, Huesing JE, Peschke VM, Eddy R, editors. Fall Armyworm in Asia: A Guide for Integrated Pest Management. Mexico City: CIMMYT (2021) 21-57.

27. Fang PX, Belton B. Maize and pigeon pea production, profitability, and tied credit in Southern Shan State. Research Paper. Feed the Future Innovation Lab for Food Security Policy, Michigan State University. (2020) 173. <https://www.canr.msu.edu/resources/maize-and-pigeon-pea-production-profitability-and-tied-credit-in-southern-shan-state> [accessed June 18, 2024].

28. Ministry of Agriculture, Livestock and Irrigation, Myanmar. Myanmar Agriculture Sector in Brief. Nay Pyi Taw: MOALI. (2020).

29. Moungsree S, Neamhom T, Polprasert S, Patthanaissaranukool W. Carbon footprint and life cycle costing of maize production in Thailand with temporal and geographical resolutions. *Int J Life Cycle Assess*. (2023) 28:891-906. doi: 10.1007/s11367-022-02021-4

30. Teerakul N, Kaewmanee P. Technical efficiency of maize production in Thailand. *Khon Kaen Agri J*. (2021) 49:595-608. doi: 10.14456/kaj.2021.53

31. Watcharasakonpong N, Thiengburanathum P. Measuring the technical and scale efficiency of maize production in Thailand: the case of Mae Chaem District, Chiang Mai. *J Econ Sustain Develop*. (2016) 7:169-181.

32. Fujisao K, Khanthavong P, Oudthachit S, Matsumoto N, Homma K, Asai H, et al. A study on the productivity under the continuous maize cultivation in Sainyabuli Province, Laos I. Yield trend under continuous maize cultivation. *Field Crops Res*. (2018) 217:167-171. doi: 10.1016/j.fcr.2017.12.016

33. Lienhard P, Lestrelin G, Phanthanivong I, Kiewvongphachan X, Leudphanane B, Lairez J, et al. Opportunities and constraints for adoption of maize-legume mixed cropping systems in Laos. *Int J Agri Sustain*. (2020) 18:427-443. doi: 10.1080/14735903.2020.1792680

34. Dorl P. Maize production and marketing in Leuk Daek District, Kandal Province, Cambodia. Research Working Paper Series. Mekong Institute, 4/2014. (2014). <https://mekonginstitute.org/maize-production-and-marketing-in-leuk-daek-district-kandal-province-cambodia/> [accessed June 18, 2024].

35. Pham VD, Napasintuwong O, Nguyen TK. An economic analysis between GM and non-GM maize production in Southern Vietnam. In: Proceedings of the 9th ASAE International Conference. 2017 Jan 11-13; Bangkok, Thailand. Asian Society of Agricultural Economics. (2017) 1081-1092. doi: 10.22004/ag.econ.284831

36. International Monetary Fund. Data from: World Economic Outlook Database. (2023). <https://www.imf.org/en/Publications/WEO> [accessed June 18, 2024].

37. United Nations Treasury. Data from the United Nations Operational Rates of Exchange. (2024). <https://treasury.un.org/operationalrates/default.php> [accessed June 18, 2024].
